# Modular microfluidic systems cast from 3D-printed molds for imaging leukocyte adherence to differentially treated endothelial cultures

**DOI:** 10.1038/s41598-019-47475-z

**Published:** 2019-08-05

**Authors:** Rodrigo Hernández Vera, Paul O’Callaghan, Nikos Fatsis-Kavalopoulos, Johan Kreuger

**Affiliations:** 1 Department of Medical Cell Biology, Uppsala University, Uppsala, Sweden; 2 Gradientech AB, Uppsala Science Park, Uppsala, Sweden

**Keywords:** Lab-on-a-chip, Cell adhesion

## Abstract

Microfluidic systems are very useful for *in vitro* studies of interactions between blood cells and vascular endothelial cells under flow, and several commercial solutions exist. However, the availability of customizable, user-designed devices is largely restricted to researchers with expertise in photolithography and access to clean room facilities. Here we describe a strategy for producing tailor-made modular microfluidic systems, cast in PDMS from 3D-printed molds, to facilitate studies of leukocyte adherence to endothelial cells. A dual-chamber barrier module was optimized for culturing two endothelial cell populations, separated by a 250 μm wide dividing wall, on a glass slide. In proof-of-principle experiments one endothelial population was activated by TNFα, while the other served as an internal control. The barrier module was thereafter replaced with a microfluidic flow module, enclosing both endothelial populations in a common channel. A suspension of fluorescently-labeled leukocytes was then perfused through the flow module and leukocyte interactions with control and TNFα-treated endothelial populations were monitored in the same field of view. Time-lapse microscopy analysis confirmed the preferential attachment of leukocytes to the TNFα-activated endothelial cells. We conclude that the functionality of these modular microfluidic systems makes it possible to seed and differentially activate adherent cell types, and conduct controlled side-by-side analysis of their capacity to interact with cells in suspension under flow. Furthermore, we outline a number of practical considerations and solutions associated with connecting and switching between the microfluidic modules, and the advantages of simultaneously and symmetrically analyzing control and experimental conditions in such a microfluidic system.

## Introduction

Microfluidic systems for studies of cell behavior are increasingly utilized in biomedical research^1,2^, and such systems are well suited for generating *in vitro* models of blood vessels, and for studying interactions of blood cells under flow with the endothelial cells that line such vessels^3–5^. Several assays have been developed for studying the adherence of leukocytes to the surface of endothelial cells^6–8^ or to extracellular matrix molecules^9–15^. Most microfluidic assay systems are created by bonding a PDMS chip containing fluidic channels to a glass substrate. Prior to bonding, fluidic inlets and outlets are typically created by mechanically punching holes to access the start and end positions of channels within the chip. The PDMS chips are cast from molds that are generally produced by a rather elaborate and expensive process involving photolitography that typically requires a cleanroom, specialized equipment and training, which present both practical and technical obstacles to many researchers.

3D-printing has relatively recently become accessible to many laboratories, and biologists and chemists are increasingly using 3D printing to create their own assays for the analyses of cells and macromolecules^16–19^. The process whereby novel assays are developed is iterative, and one of the many advantages of 3D printing is that it radically speeds up the process of going from idea to a first prototype. Another important advantage of 3D printing is that it often allows for greater geometrical complexity, such that molds for PDMS casting with combinations of structures of different heights easily can be generated in a single 3D print. A number of microfluidic systems have been created directly by 3D-printing or by creating molds using 3D-printing for PDMS casting^20–23^, and innovative applications of 3D printing will continue to expand the possibilities for user-customized assay development within the life sciences.

Here we present a strategy based on a set of 3D-printed tools and molds for PDMS casting that allow researchers to build a modular system for imaging of the adherence of the Jurkat cell line (a commonly used leukocyte model for T cell leukemia) from a single population to differentially treated endothelial cell cultures. A barrier module is reversibly attached to a glass microscope slide; two adjacent endothelial cultures are seeded, and in a proof-of-principle experiment one is exposed to the inflammatory cytokine tumor necrosis factor α (TNFα), while the other untreated culture serves as an internal control. The barrier module is exchanged for a flow module to permit the perfusion of a single leukocyte population over both endothelial conditions and images are captured by time-lapse microscopy. The devices and method presented here offer a number of advantages to researchers seeking an *in vitro* model of immune or cancer cell interactions with endothelial cells in a setting that is comparable to small blood vessels. The increasing availability of 3D-printing services permits researchers without design or manufacturing expertise to acquire molds and produce their own devices at low cost; equally, researchers with experience in this field can customize the modules for adaption to their specific applications.

## Results and Discussion

### System design

**Figure 1:**
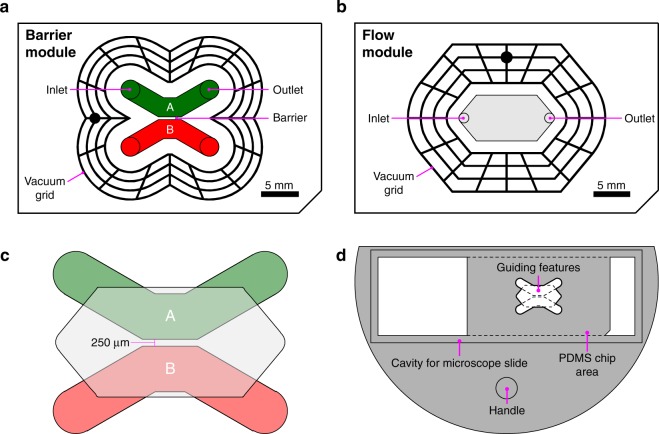
System design. (**a**) Design of the mold for the barrier module. The green and red fields correspond to channels used for cell seeding separated by a 250 μm wide barrier, and the black lines correspond to a network of vacuum channels that when connected to a vacuum source was used to attach the chip to a glass slide. (**b**) Design of the mold used to create the flow module. (**c**) Overlap of the cell seeding channels of the barrier module and the perfusion chamber of the flow module. After module exchange, cells within the seeding channels A and B (green and red) will fit within the perfusion chamber of the flow module (light grey). (**d**) Design of the alignment tool.

The system presented here was designed to study interactions between a single population of leukocytes and two distinctly treated endothelial cell populations, namely TNFα-treated and control. To achieve this, two modules were designed: a barrier module (Fig. 1a) and a flow module (Fig. 1b). The barrier module permitted adjacent seeding of the two endothelial populations separated by a 250 μm wide barrier, which ultimately allowed for imaging of both populations in a single field of view. The flow module was designed to simultaneously perfuse a single population of leukocytes over both endothelial populations, facilitating concurrent imaging of leukocyte interactions with TNFα-treated and control endothelial cultures. The z-axis height of the culture chambers in the barrier module and in the flow chamber were designed to be 200 µm. Vacuum grids^24,25^ were placed around the centrally positioned fluidic channels of both the barrier and flow modules to enable a reversible attachment to glass substrates (Fig. 1a,b), these grids were designed to have a z-axis height of 100 µm.

To facilitate module switching and ensure precise positioning of the flow module relative to the barrier module (Fig. 1c) (and consequently the two endothelial cell cultures) a 3D-printed alignment tool was designed (Fig. 1d). The alignment tool consisted of a semi-circular plate designed to fit inside a 100 mm Petri dish with a holder to accommodate a microscope slide. The holder was designed with an opening in the middle matching the key features present in both modules (Fig. 1d). The alignment tool ensured that each PDMS module was properly aligned with its corresponding features in the holder before applying vacuum to securely attach it to the glass slide. To aid in the visualization of the 3D printed molds and the alignment tool, illustrations prepared using Fusion 360 CAD software are presented in Supplementary Fig. S1.

3D printed molds and modular microfluidics have been described in a number of previous studies^26–30^; however, the modularity described by Lee *et al*.^28^ predominantly demonstrates the utility of laterally combining and vertically stacking distinct microfluidic systems, which are intended for use in diagnostic applications. Bachmann *et al*.^26^ produce PDMS devices from 3D printed molds for the purpose of culturing endothelial cells in fibrin gels, but the microfluidic channels in this system are generated by post processing techniques rather than cast from 3D printed molds. The modular system described here facilitates distinct microfluidic operations to be performed on the same pair of HUVEC populations using relatively simple PDMS devices that are entirely derived from molds printed from a widely accessible type of 3D printer. Sun *et al*.^29^ present an innovative chemical-based method for increasing the resolution of microfluidic systems cast from 3D-printed molds, which aims to increase the availability of high resolution systems for researchers who lack access to expensive microfabrication techniques. The systems and applications described herein also aim to demonstrate that researchers from cell biology fields can adopt and apply microfluidic principles to their studies without the need to gain expertise in microfabrication and without the need for access to expensive facilities or equipment.

### Printer calibration

When using bottom-up stereolithography 3D printers, objects are commonly oriented to print at an angle to minimize the cross-sectional area of each layer, which requires the use of supporting structures. As PDMS surface smoothness is critical for its correct bonding to glass, we first analyzed the effect that the orientation used for 3D printing of the mold had on the smoothness of the PDMS chips cast in it. Clearly, PDMS chips cast from molds that were printed parallel to the 3D printer build platform had a smoother surface appearance and bonded better to glass than those cast from molds printed at a tilted angle (see Supplementary Fig. S2). Next, the 3D printer’s resolution was tested. The laser spot size of the Form 2 printer is 140 µm so to assess the resolution of PDMS chips, 3D molds were designed with round and square features ranging from 125 μm to 1 mm. Microscopic inspection of such features in PDMS chips cast from these molds revealed that the resolution limit for these features was between 250 and 500 μm (see Supplementary Fig. S2).

**Figure 2:**
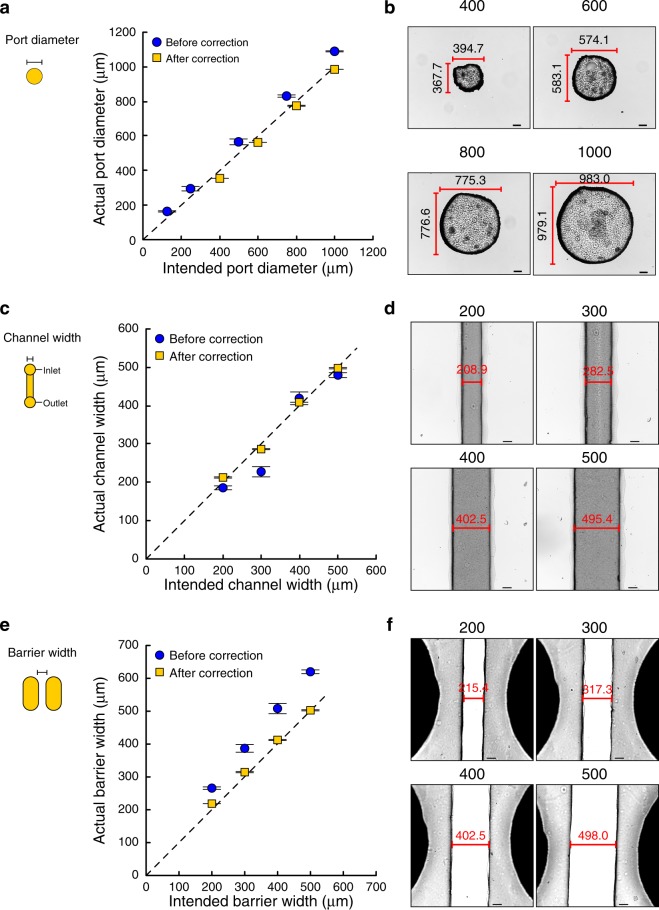
Calibration process. An iterative calibration process was used to find the appropriate design dimensions to create ports (**a,b**), channels (**c,d**) and barriers between cell culture chambers (**e,f)** in PDMS using 3D-printed molds. (**b,d,f**) Representative images of PDMS structures formed after adjustment of the mold CAD files based on the obtained correction factors; the measured feature lengths are indicated in the images and the intended feature lengths shown above each panel (all units = μm). It was not possible to consistently create barriers that were narrower that 200 μm.

Calibration experiments were carried out to characterize the precision of the 3D printer. Briefly, mold structures in the CAD files were compared to the sizes of corresponding structures in the created PDMS chips, focusing on features relevant to microfluidic applications: inlet/outlet ports (Fig. 2a), channels (Fig. 2c), and barriers (Fig. 2e). Images of the features were captured and the actual dimensions of structures were measured using ImageJ software and then plotted against the intended dimensions. Examples of these plots are presented in Fig. 2 as the blue data points representing dimensions ‘Before correction’. Linear regression analysis was performed and the equations of the lines were used to define a correction factor to be used when assigning the dimensions in CAD drawings of molds. The following correction factors were determined: for mold pillars (corresponding to inlet/outlet ports): CAD drawing dimension = (0.876 × desired PDMS dimension) + 34.456; for PDMS channels: CAD drawing dimension = (0.827 × desired PDMS dimension) + 12.310; for PDMS barriers CAD drawing dimension = (0.865 × desired PDMS dimension) + 137.159. The application of these correction factors ensured that the actual dimensions of PDMS structures more closely matched the intended dimensions (Fig. 2b,d,f).

The minimum layer thickness for structures printed with the Form 2 stereolithography printer is 25 µm, defined by the stepper motor that vertically moves the build platform. We assessed the height of structures in PDMS chips cast from 3D printed molds and determined that the standard deviation for the actual heights of structures intended to be 100 µm or 200 µm (i.e. the height used in the barrier and flow modules) were below the printer’s minimum layer height of 25 µm; therefore, no correction factor was applied to the z-axis dimensions (see Supplementary Fig. S3).

### Seeding and differential treatment of distinct endothelial cell populations in the barrier module

**Figure 3:**
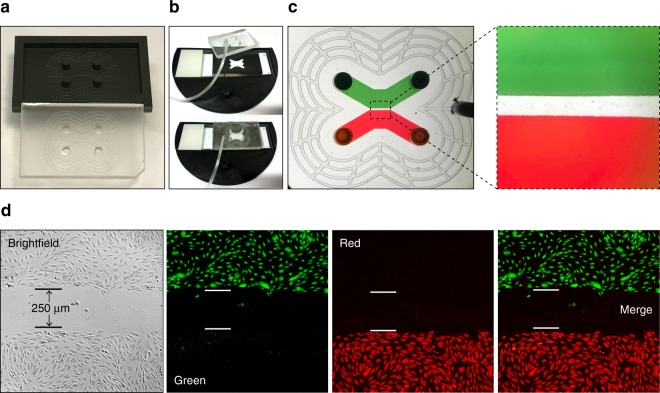
Production of the barrier module for seeding of two endothelial cell populations separated by a 250 µm barrier on a glass slide. (**a**) The barrier module was cast in PDMS from a 3D-printed mold. (**b**) Precise positioning of the barrier module to a glass slide using a 3D-printed alignment tool and attached using vacuum. (**c**) The barrier module vacuum-attached to a glass slide with its cell seeding channels filled with either a red or a green dye. (**d**) Endothelial cell populations grown in the barrier module overnight and thereafter stained for 4 h in their respective channels with Celltracker green or Celltracker red dyes, and imaged using a fluorescence microscope.

The barrier module mold produced a device consisting of two 2.5 mm-wide seeding channels (200 µm in height) separated by a 250 µm barrier (Fig. 3a,b). Ports (2.5 mm in diameter and 3 mm in height) were placed in both ends of the channels to allow cell seeding using a micropipette, and a vacuum grid (200 µm wide and 100 µm tall channels) surrounded the fluidic channels. A barrier module was positioned on a glass slide with the aid of the alignment device (to later on facilitate accurate module switching) and then vacuum attached (Fig. 3b). Red or green dye were in initial tests added to the seeding channels and confirmed that the vacuum-assisted attachment of the module resulted in a tight seal to the glass slide with no leakage across the barrier between the channels (Fig. 3c).

Next, experiments with cells were conducted. Human umbilical vein endothelial cells (HUVECs) were added to each seeding channel of the barrier module and allowed to attach, and spread overnight. The following day the HUVEC populations were stained with fluorescent dyes (one side with Celltracker green and the other side with Celltracker red) and washed in the module. Subsequently the module was removed and the cells were imaged using brightfield and fluorescence microscopy, which revealed confluent, differentially stained populations that were indeed separated by a 250 µm wide gap (Fig. 3d).

The incorporation of a barrier into a microfluidic model of a placental barrier has previously been employed by Mandt *et al*.^31^. In that system, the barrier was established using 2-photon polymerization to polymerize gelatin within the device. Cells are cultured on either side of the barrier, which represents a basement membrane and is essentially an integral component of the model system. In contrast, the barrier that we employ is simply intended to physically separate two differentially treated cell types and is not required to integrate with the cultured cells; additionally, the modular approach adopted means that the barrier is removable and exchangeable. Importantly, given the small division that the barrier creates between the differentially treated HUVEC populations, both control and treated populations can be simultaneously imaged in a single frame of view.

### Flow module attachment and leukocyte perfusion on distinctly treated endothelial cell populations

**Figure 4:**
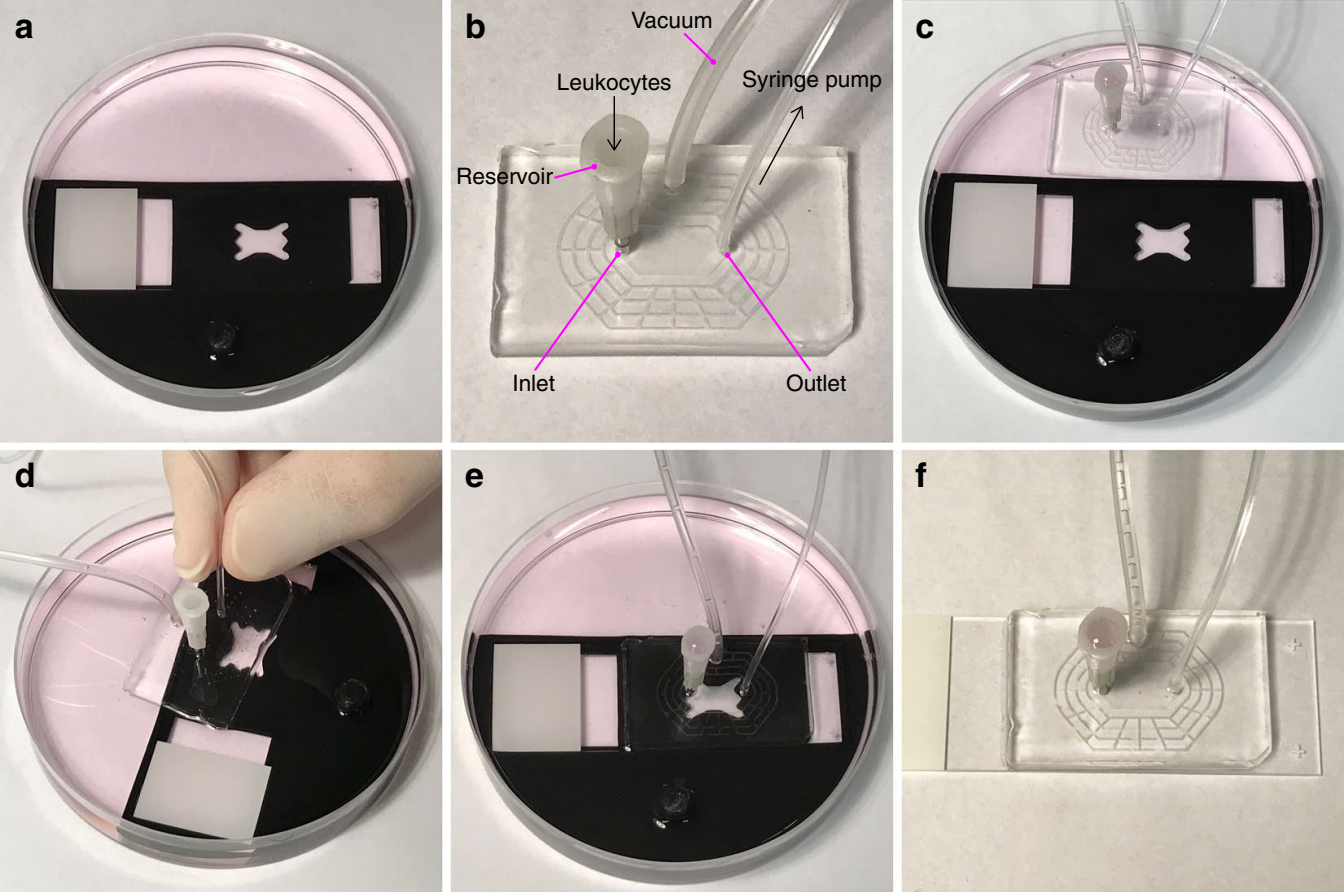
Under-medium module exchange procedure. (**a**) The glass slide on which the two endothelial cell populations were seeded was submerged in a Petri dish containing cell medium and positioned in the alignment tool. (**b**) The flow module was connected to a vacuum source, and the outlet is connected to a syringe containing culture medium. A reservoir for leukocyte loading was inserted in the inlet. (**c**) The flow module was lowered into the Petri dish containing the endothelial cell slide positioned in the alignment tool, vacuum was applied and the perfusion chamber and leukocyte reservoir was filled with cell culture medium via the outlet tubing, to confirm that the system was not leaking. (**d**) The vacuum was switched off and the flow module was detached from the bottom of the Petri dish. While still submerged, the flow module was positioned above the glass slide, and aligned with the endothelial cell populations using the alignment tool. (**e**) The flow module was carefully pressed in place and vacuum applied to seal it to the glass slide. (**f**) The flow module and attached endothelial slide was removed from the alignment tool in the Petri dish and dried before transfer to a microscope for imaging.

In a new set of experiments, HUVEC populations were cultured overnight in the barrier module. Confluence was visually confirmed, where after one endothelial cell population was treated with TNFα, while the other served as a control population. Simultaneously, both HUVEC populations were labeled with Celltracker red. In parallel, a suspension of Jurkat leukocytes was stained with Celltracker green. At the end of the treatment, the TNFα and control solutions were removed from the HUVEC populations in the barrier module and cells were extensively washed with culture medium. The barrier module was then carefully peeled away and the glass slide placed in the alignment tool and submerged in a 100 mm Petri dish containing cell culture medium (Fig. 4a). Prior to attaching the flow module to the glass slide, the inlet, outlet and vacuum ports were connected (Fig. 4b). Specifically, the outlet from the flow module was via tubing connected to a 1 ml syringe half-filled with cell medium, while the inlet was connected to a leukocyte reservoir. The complete flow module was then submerged into the Petri dish adjacent to the HUVEC slide in the alignment tool (Fig. 4c). The vacuum seal and inlet-to-outlet connections of the flow module were tested by pressing the module to the bottom of the dish and applying vacuum. The flow chamber and leukocyte reservoir were then filled with culture medium via the outlet tubing connected to the syringe. No loss of medium from the reservoir confirmed that the module was properly sealed and connected (Fig. 4c). The vacuum was turned off and while still submerged in medium the flow module was floated above the glass slide (on which the two endothelial populations were positioned) in the alignment tool (Fig. 4d). Exchanging the modules under medium eliminates the risk of introducing air bubbles into the microfluidic channels, and reduces the risk of cells drying out. Once correctly aligned using the alignment tool, the flow module was carefully pressed to the glass slide and attached by applying vacuum (Fig. 4e). The connected flow module and HUVEC slide were then removed from the Petri dish (Fig. 4f) and excess medium on the slide wiped away before placing the assembled flow module onto the microscope stage.

**Figure 5:**
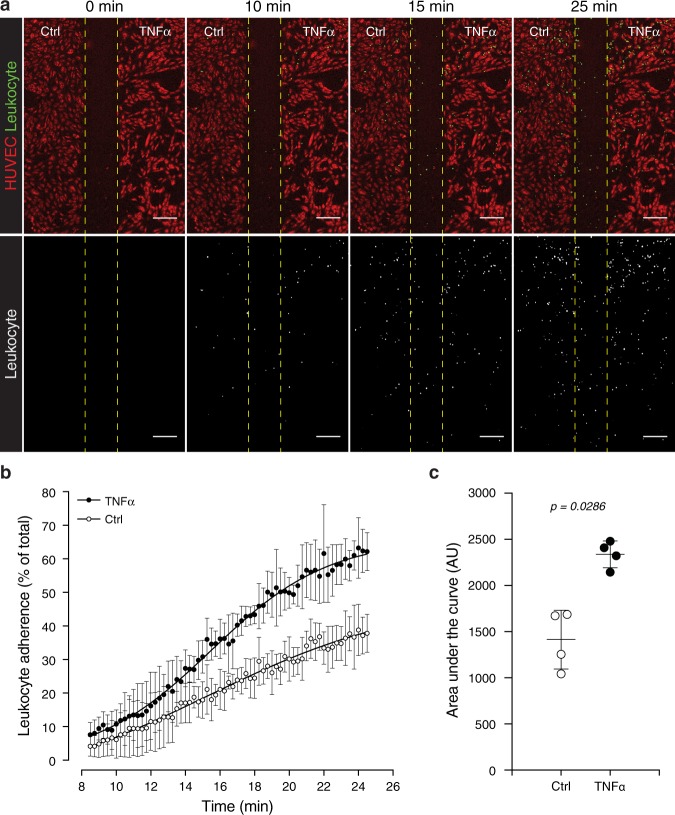
Leukocytes preferentially attach to TNFα-activated endothelial cells. (**a**) Celltracker red staining illustrates HUVECs on the Ctrl and TNFα-treated sides following removal of the barrier module. Time-lapse images in the flow module reveals attachment of Celltracker green-stained leukocytes to HUVECs over time. The lower panels present leukocyte fluorescence only (grayscale) to aid in visualizing their distribution. (**b**) Relative percentage of leukocytes attached to Ctrl and TNFα-activated HUVECs over time. (**c**) Area under the curve (AUC) analyses of leukocyte attachment to Ctrl and TNFα-activated HUVECs. Data-points in (**b**,**c**) represent mean ± standard deviation for n = 4 independent experiments. Statistical significance was assessed using the non-parametric Mann-Whitney test to compare the ranks of the AUC values (Original magnification: (**a**) 5x objective; Scale bar = 200 μm).

Endothelial cells and the Jurkat leukocyte cell line were visualized using an inverted fluorescence microscope with a 5x objective. The slide was positioned such that the gap produced by the barrier between the TNFα-treated and control HUVEC populations was centralized in the field of view, with the edge of the leukocyte inlet just outside the field of view to ensure that the earliest point of leukocyte inflow was imaged. The outlet was connected to a pump in withdrawal mode. The Celltracker green-labeled leukocyte suspension was added to the medium in the leukocyte reservoir and time-lapse images of leukocytes flowing over and attaching to Celltracker red-labeled endothelial cells were captured. Image analysis revealed increased adherence of leukocytes to TNFα-activated HUVECs over time, compared to untreated controls (Fig. 5a–c and Supplementary Video V1), in line with previous results^8^. Microfluidic devices that enable quantitative image analysis of a greater range of leukocyte interactions with endothelial cells, including leukocyte migration following rolling and adherence, have been developed elsewhere. For example, Lamberti *et al*.^7^ use SU-8 photoresist lithographically patterned onto silicon wafers to produce a multi-layered mold. This type of mold is not trivial to fabricate, and relies on access to the relatively specialized (and expensive) environment of a clean-room facility, but considerably smaller structures can be resolved using this process. In contrast, the molds for the modular system presented here are fabricated by the relatively simpler and less expensive process of 3D printing. Multi-layer features are readily included in the 3D printed molds due to the layer-by-layer manner in which the parts are printed. As mentioned, in the Lamberti *et al*. device it is possible to analyse additional parameters including transmigration of leukocytes across the cultured endothelium. However, while the impressive ‘bioinspired’ geometry of the vascular networks in the Lamberti *et al*. device represent a faithful model of the *in vivo* environment, it likely produces a more complex dataset for image analysis than that derived from our system. Additionally, the ability to analyze adherence of a common leukocyte suspension to both control and treated HUVEC populations in the same field of view ensures that every experiment conducted in the system described here includes its own internal control.

Celltracker red stained endothelial cells were also imaged at higher magnification and fluorescence intensity was visualized using a 16-color spectrum (see Supplementary Fig. S4). This illustrated that the degree of endothelial confluence could be under-appreciated at low magnification and when using a monochromatic representation of Celltracker red fluorescence. This is due to the relatively lower concentrations of dye in the extremities of the cell due to reduced cell height and therefore reduced cytoplasmic volume (see Supplementary Fig. S4). Additionally, at higher magnification and with shorter time-lapse intervals the system could be used to study leukocyte deceleration (see Supplementary Fig. S5 and Video V2).

To determine the effect the evacuation of the vacuum grid had on dimensions of the flow module we introduced a suspension of fluorescent microspheres (2 µm in diameter) diluted in a soft, easily deformable agarose gel into the flow chamber. The spatial distribution of microspheres along the entire z-axis in a central location of the chamber was imaged by confocal microscopy, with vacuum off and on. Image analysis of composite images revealed that evacuating the vacuum grid had a minimal effect on the intended geometry of the flow chamber (see Supplementary Fig. S6).

### Assessing leukocyte distribution, laminar flow, and the potential for transverse diffusion in the flow module

To assess potential regional differences in the adherence of leukocytes within the flow module, images of leukocyte fluorescence from the final time-point of three independent experiments were stacked together and the area of leukocyte fluorescence from regions of interest along the vertical (top to bottom) and horizontal axes (left to right) were measured (see Supplementary Fig. S7). The degree of leukocyte attachment decreased along the vertical axis from the top to the bottom of the flow module. This is likely due to the gradual depletion of leukocytes from the flow suspension as they bind to the endothelial surface. The degree of attachment along the horizontal axis was lowest at the left and right extremities, which is predicted by the laminar flow of the leukocyte suspension in this module (see Supplementary Fig. S7). These observations emphasize the advantage of simultaneously imaging leukocyte adherence in the control and TNFα-treated HUVECs under the symmetrical conditions afforded by the modular microfluidic systems described here. Furthermore, it illustrates the need to account for regional differences in leukocyte distribution within similar microfluidic systems, and highlights the importance of analyzing identically located regions of interest, when control and treatment experiments are performed in separate microfluidic devices. To visually inspect that the paths followed by objects in the flow module conformed to laminar flow patterns we manually tracked dye particles passing through the central location of the flow module and observed that, as predicted, they travelled along the vertical access of the flow module in parallel to each other (Supplementary Fig. S8b,c and Video V3). Next, we constructed a COMSOL simulation to determine to what degree solutes from the treated side of the chamber (in particular the TNFα cytokine) would diffuse to the control side of the chamber for the duration of the leukocyte flow experiments. The results from this modelling suggested that the laminar flow path, instated during the leukocyte flow experiments, creates a barrier that effectively limits diffusion of solutes from one side of the chamber to the other (see Supplementary Fig. S8). Therefore, the risk of a substantial transfer of molecules between the two cell populations within the flow chamber (which could lead to confounding paracrine signaling events) is deemed negligible.

## Conclusion

The modular microfluidic system presented here, created using a set of 3D-printed tools and molds for PDMS casting, enables high-resolution imaging and analyses of leukocyte adherence from a single perfused population onto two endothelial cell cultures. A major advantage of the current system is that it allows simultaneous monitoring of both treated and control endothelial cells in the same field of view. Furthermore, the method described for under-medium microfluidic module switching enables researchers to seed, treat and analyze distinct cell populations by sequential application of modules with different functionalities. While the present system was optimized for imaging of leukocyte adherence to differentially treated endothelial cultures, similar devices can readily be customized for other applications.

## Materials and Methods

### 3D-printing

All 3D-printed objects were drawn using Autodesk Fusion 360 (Autodesk). Molds for PDMS casting were printed with a Form 2 printer (Formlabs, Somerville, MA, USA) using black resin and 25 µm thick layers. The alignment tool was printed using an Ultimaker 3 Extended printer (Ultimaker B.V., Geldermalsen, The Netherlands) equipped with a 0.4 mm nozzle using black polylactic acid filament and 150 µm layers. Illustrations of the barrier module mold, the flow module mold and the alignment tool are presented in Supplementary Fig. S1. The CAD (.step) and stereolitography (.stl) files for all of the printed pieces are appended to the manuscript as supplementary information in the compressed file named Barrier_Flow_Alignment CAD and STL files.

### PDMS chip fabrication

Microfluidic chips were cast using 1:10 PDMS (Sylgard 184, Sigma-Aldrich Sweden AB, Stockholm, Sweden). The PDMS modules were reversibly bonded to glass slides by connecting the vacuum grid to a vacuum source. For the printer calibration experiments, PDMS chips were bonded to glass after plasma treatment.

### Cell culture

Human umbilical vein endothelial cells (ATCC, Manassas, VA, USA) were cultured in endothelial basal medium 2 (EBM2; BioNordika, Stockholm, Sweden) conditioned with the microvascular BulletKit (MV; BioNordika) in cell culture flasks pre-coated with 0.1% gelatin. Jurkat leukocytes were cultured in suspension in Roswell Park Memorial Institute (RPMI) medium (Gibco, Thermo Fisher Scientific) supplemented with 10% fetal bovine serum (FBS; Gibco, Thermo Fisher Scientific).

### Seeding of HUVECs into the dual-chamber barrier module

The barrier module was first positioned on a Superfrost glass microscope slide (ThermoFisher Scientific) using the alignment tool. The barrier module was attached to the glass slide by inserting 1.5 mm diameter tubing (Tygon; Bergman Labora AB., Danderyd, Sweden) into the vacuum port and connecting it to a vacuum source. To clean and wet the surfaces of the fluidic channels in the PDMS modules, 50 μl of 70% ethanol was pipetted into each inlet and withdrawn through each outlet. For gelatin coating of the barrier module 50 μl of 0.1% gelatin was pipetted into each inlet, withdrawn through each outlet and discarded, following which an additional 50 μl of 0.1% gelatin was added to each chamber and incubated for 30 min at room temperature. Prior to the addition of HUVEC suspensions, the gelatin solution was removed and the chambers were washed twice with 50 μl of EBM2-MV medium, and the chambers then filled with an additional 50 μl of EBM2-MV medium. A HUVEC suspension of 2 × 10^6^ cells/ml was prepared in EBM2-MV medium and 50 μl was added to each chamber’s inlet, and there after withdrawn through the outlet and discarded. This process was repeated, after which a final 50 μl of the HUVEC suspension was added to each chamber. This method for seeding HUVECs ensured an even and thorough distribution of cells against the barrier and throughout the chamber. The inlets and outlets were sealed using Parafilm (Sigma-Aldrich Sweden AB) that was weighted in place to reduce evaporation of culture medium. HUVECs were cultured overnight (37 °C, 5% CO_2_) and confluence was assessed using a standard cell culture laboratory microscope.

### Attachment of the flow module and leukocyte perfusion over control- and TNFα-treated HUVECs

Confluent HUVEC populations cultured using the barrier module were treated for 4 h with or without 1 ng/ml TNFα (PeproTech, Rocky Hill, NJ, USA) in EBM2 containing 1% FBS and 5 μM of Celltracker red (ThermoFisher Scientific). Jurkat leukocytes were stained with 1 μM Celltracker green (ThermoFisher Scientific) and shortly before perfusion a suspension of 1 × 10^5^ leukocytes/ml was prepared in RPMI, 10% FBS. Following the 4 h treatment the TNFα and control solutions were removed from the barrier module and the HUVEC populations were washed twice with 50 μl of EBM2, 1% FBS. The barrier module was then peeled away and the slide was submerged in a 100 mm Petri dish containing DMEM, 10% FBS. Prior to attaching the flow module to the differentially treated HUVEC populations, 2 mm diameter tubing (Tygon) was inserted into the vacuum port of the flow module and connected via a stopcock valve and vacuum flask to a vacuum source. The outlet from the flow module was connected to a 1 ml syringe (Microlance, VWR, Stockholm, Sweden) via 1.5 mm diameter tubing (Tygon), which had been filled (approximately 500 μl) with RPMI, 10% FBS medium. A leukocyte reservoir was connected to the inlet of the flow module. The reservoir consisted of a 16-gauge needle (Microlance) whose shaft had been shortened to a length of 5 mm. The perfusion device with its three connected ports (Fig. 4b) was submerged into the 100 mm diameter Petri dish containing the HUVEC slide (Fig. 4a,c) and DMEM, 10% FBS. The vacuum seal and inlet-to-outlet connection was first tested by manually pressing the perfusion chamber to the bottom of the Petri dish, evacuating the vacuum grid by opening the stopcock valve and then filling the flow chamber and the leukocyte reservoir via the outlet tubing using the syringe. The selected flow module was used for experiments if no loss of the medium from leukocyte reservoir was observed. The vacuum was then turned off, but the flow module was kept submerged, ensuring that the reservoir, flow chamber and vacuum line contained medium, which reduce the risk of trapping air bubbles in the system. The flow module was then floated above the HUVEC populations and correctly positioned using the alignment tool (Fig. 4d,e). The module was carefully pressed to the glass slide, after which the vacuum was opened and the grid was evacuated (Fig. 4e). The connected flow module and HUVEC slide were removed from the Petri dish (Fig. 4f) and excess medium on the slide was carefully soaked off using a tissue before placing them on the microscope stage.

Celltracker red fluorescence from HUVECs was visualized using an Axiovert 200 M fluorescence microscope (Zeiss, Jena, Germany) with a 5x objective. The slide was positioned such that the gap produced by the barrier between the TNFα and control treated populations was centralized in the field of view and the edge of the leukocyte inlet was immediately outside of the field of view. Once correctly positioned an image of the Celltracker red HUVEC fluorescence was captured. The syringe connected to the outlet was secured in a syringe pump (Harvard Apparatus Model 22 Syringe Pump 980532, Holliston, MA, USA) set in withdrawal mode to generate a flow of 4 μl/min. One hundred microliters of the Celltracker green stained Jurkat leukocyte suspension was added to the medium in the filled leukocyte reservoir; which prevented air bubbles from being introduced to the the flow module. Time-lapse images of Celltracker Green stained leukocytes were captured using AxioVision software (Zeiss). After the final leukocyte image was acquired a second image of the Celltracker red stained HUVEC populations was collected to assess possible device movement during image capturing.

### Quantitative image analysis of leukocyte attachment to control- and TNFα-treated HUVECs in the flow module

The ImageJ rolling ball radius background subtraction and despeckle processing functions were applied to the fluorescence from Celltracker red labeled HUVECs and the time-lapse series of fluorescence from Celltracker green labeled leukocytes. The ImageJ rectangular selection tool was used to designate individual regions of interest (ROI) of equal size in the Ctrl- and TNFα-treated HUVEC populations. These ROI were overlaid on the time-lapse series of leukocyte attachment and the number of Celltracker green stained leukocytes per frame in Ctrl- and TNFα-treated HUVECs was analyzed using the ImageJ particle analysis plugin. Additionally, the total number of HUVECs in the Ctrl- and TNFα-treated ROI were counted and recorded using the ImageJ multipoint tool. For each frame the number of leukocytes per HUVECs in the Ctrl- and TNFα-treated ROIs were expressed as a percentage of the total number of leukocytes in both ROIs at the final time-point. These values were plotted over time and the area under the curve for the resulting plots were calculated.

### Imaging of the calibration structures, and the barrier and flow module

Images for port-, channel-, and barrier-calibrations were taken using an Axiovert 200 M fluorescence microscope (Zeiss). Measurements were performed with the AxioVision software (Zeiss). Low magnification images of the different systems containing dyes were taken with an Iphone SE coupled to a Nikon SMZ1270 stereomicroscope (Nikon, Tokyo, Japan) using an adaptor, as previously described^18^.

## Supplementary information

Supplementary Materials and Methods, Figure Legends and Figures

Leukocyte attachment to HUVECs in the flow module

Leukocyte attachment to HUVECs in the flow module (high magnification)

Manual tracking of dye particles passing through the flow module

CAD and sterolitography files

## Data Availability

The datasets generated and analyzed during the current study are available from the corresponding author on request. The CAD and stereolitography files are included as Supplementary Information in the compressed file Barrier_Flow_Alignment CAD and STL files.
