# Supplementary material for: Modular microfluidic systems cast from 3D-printed molds for imaging leukocyte adherence to differentially treated endothelial cultures
Source: Sci Rep. 2019 Aug 5;9:11321. doi: 10.1038/s41598-019-47475-z (PMC6683170; doi:10.1038/s41598-019-47475-z)
Supplement: Supplementary file 1 — Supplementary Materials and Methods, Figure Legends and Figures [file 41598_2019_47475_MOESM1_ESM.pdf]

Supplementary Information
Modular microfluidic systems cast from 3D-printed molds for imaging leukocyte adherence to differentially treated endothelial cultures

Rodrigo Hernández Vera[†, a], Paul O'Callaghan[†, a], Nikos Fatsis-Kavalopoulos[a, b], Johan Kreuger[*, a]

[a]Department of Medical Cell Biology, Uppsala University, Uppsala, Sweden

[b]Gradientech AB, Uppsala Science Park, Uppsala, Sweden

[†]Equal contribution

[*]Corresponding author. E-mail: johan.kreuger@mcb.uu.se

Supplementary Materials and Methods

Analysis of the intended and actual z-height of PDMS structures cast from 3D printed molds

To analyze the difference between the intended and actual z-height of PDMS structures cast from 3D printed molds a mold containing 9 cuboid structures with heights increasing in steps of 100 μm from 100 μm to 900 μm was designed and drawn using Fusion 360 software (Fig. S3a). Three replicates of the mold were 3D printed using the same FormLabs printer used to print the barrier and flow modules. PDMS chips were cast from the mold and three cross-sections (approximately 1 mm thick) were cut through the length of each chip using a microtome blade. Each cross-section was then mounted onto a glass slide and images of each of the PDMS wells (100-900 μm z-axis dimensions) were captured using the DIC function on a confocal microscope (LSM 700, Zeiss, Jena, Germany). The images were imported to ImageJ where the line tool was used to manually measure the actual height of each of the PDMS wells (Fig. S3b insert and c). The average of the heights measured for each well from each of the three cross-sections was determined for each mold. The average ± standard deviation for each well from the three replicate molds was plotted against the intended height, and a linear regression analysis was performed using Prism 7 software (Fig. S3d).

Image analysis of leukocyte deceleration on HUVECs in the flow module

Time-lapse images of Celltracker green leukocyte fluorescence was captured in the flow module at 5 s intervals using a 20x objective. An example of leukocyte deceleration and attachment to HUVECs was selected (Fig. S5a). In ImageJ the time-lapse sequence of interest

was isolated as a substack and the x- and y-coordinates for the front edge of the leukocyte of interest determined by manually defining ROIs using the multi-point tool (Fig. S5b). This permitted the velocity of the leukcoyte from one frame to the next to be calculated as the product of the distance travelled divided by the time interval between frames (Fig. S5c). The trajectory of the leukocyte in the time-lapse sequence of interest was also represented as a temporal color-coded projection using the ImageJ hyperstack function (Fig. S5b 0-30 s).

Effect of vacuum bonding on the height of the flow module

To assess the effect of evacuating the vacuum grid on the geometries of the flow module the volume of the flow chamber was imaged with the vacuum applied (on) and switched off. To visualize the flow chamber volume fluorescent microspheres (FluoSpheres carboxylate (580/605), Thermo Fisher Scientific) with a diameter of 2 μm were suspended in a 0.4 % solution of agarose (Top Vision Agarose, Thermo Fisher Scientific) dissolved in water. The solution was drawn into a flow module mounted on a cover glass where it polymerized as an easily deformable soft gel (Fig. S6a). The device was transferred to a confocal microscope stage (LSM 700, Zeiss, Jena, Germany) and the center of the flow module was visualized with a 20X objective using Zen imaging software (Zeiss). With no vacuum applied a z-stack from the bottom to the top of the flow chamber (as determined by the lowest and highest position in which fluorescent spheres were detected) was acquired. The pinhole was set to achieve an optical section thickness of 1 μm. The vacuum was turned on and the same z-stack acquisition settings were applied to collect a second stack of the flow chamber. The fluorescent microspheres in the z-stack acquired with the vacuum off were pseudo-colored red, and those in the z-stack acquired with the vacuum on were pseudo-colored green. Using ImageJ image analysis software the z-stacks of the x, y planes were projected into a single image, and the spatial overlap between images of microspheres acquired with the vacuum off and on were compared by merging the two images into one composite image (Fig. S6b). To assess the spatial distribution of microspheres along the z-axis of the flow module the z-stacks were resliced using ImageJ such that the z, x axis were presented in a y-stack, which was projected into a single z, x plane. The projected z, x stacks of microspheres imaged with the vacuum off and on were merged to create a composite image which allowed for the vacuum-mediated displacement of microspheres to be visualized (Fig. S6c). Individual microspheres in the upper (n=35) and lower (n=32) positions of the z, x projected stack acquired with the vacuum off were identified as regions of interest (ROIs), and the position of the same microspheres were identified as ROIs in the z, x projected stack acquired with the vacuum on. The co-ordinates for each of the ROIs were exported to Excel and the formula for the distance between two-points ($\sqrt{}(x_2-x_1)^2+(y_2-y_1)^2$) was applied to calculate the

displacement of microspheres as a result of evacuating the vacuum grid. The displacement for each microsphere analyzed in the upper and lower part is presented as a percentage of the channel height (200 μm) using a scatter dot plot prepared using Prism 7 software (Fig. S6d).

Image analysis of leukocyte distribution in the flow module

The final time-lapse images of Celltracker green leukocyte fluorescence, from three independent adherence experiments (also analyzed in Fig. 5), were stacked into a single image. Ten rectangular regions of interest (ROIs) were defined from top to bottom along the vertical axis of the image on the Ctrl side of the device, and a further 10 were defined on the TNFα side; these 20 regions collectively represented the entire imaged area of the flow module. Additionally, 10 rectangular ROIs were defined from left to right (as indicated in along the horizontal axis of the image (i.e. 5 ROIs on the Ctrl side and 5 ROIs on the TNFα-treated side); these also collectively represented the entire imaged area of the flow module (Fig. S7a). A threshold for the Celltracker green leukocyte fluorescence was set for the entire image and the area of fluorescence in each of the defined ROIs was measured using ImageJ. These values were presented as a percentage of the total area of fluorescence from all ROIs along the vertical (Fig. S7b) or horizontal (Fig. S7c) axes, respectively. Two examples of the ROIs selected on the vertical axis are illustrated by the pink and yellow frames, and one example of the ROIs selected on the horizontal axis is illustrated by the cyan frame (Fig S7a).

Laminar flow properties and analysis of potential transverse diffusion in the flow module

To assess the flow patterns within the flow module a black dye was diluted in water to yield a suspension containing easily distinguishable dye particles. The dye suspension was drawn into the flow module and filled the chamber (Fig. S8a). The flow module was placed on the stage of the Axiovert 200M microscope (Zeiss) and the central position across the midline of the chamber was viewed using the live feed from the AxioVision imaging software, which was recorded in real-time using screen capture software. As for the leukocyte experiments, a withdrawal flow with a rate of 4 μl/min was applied to the chamber. The screen captured videos of dye particles passing through the flow module were imported to ImageJ as image stacks and individual particles were tracked using the Manual Tracking ImageJ plugin over 100 frames. The complete track (dot-and-line) for each tracked particle was recorded and presented overlaid on the first frame of the image sequence (Fig. S8b), and for clearer visualization were also presented on a black background (Fig. S8c).

To determine the risk of transverse diffusion in the flow module (i.e. whereby solutes from one side of the module would diffuse to the opposite side) a finite elements model of the

flow module was constructed in COMSOL to simulate the Ctrl and TNFα-treated HUVEC populations. The model aimed to simulate a hypothetical situation where a factor such as TNFα was constantly released at a fixed concentration from the TNFα treated HUVECs. This scenario was modeled to depict the release of TNFα bound to the surface of the treated endothelium or the induced release of a similar cytokine. A flow rate of 4 μl/min was applied to the model and the diffusion coefficient for the simulated TNFα was obtained from a mathematical approximation published by Goodhill et al.[1] After 25 mins of simulated flow in the model (i.e. the same duration as the leukocyte experiments) the average concentration of TNFα on the Ctrl and TNFα sides of the model were calculated (Fig. S8d). This demonstrated that 98.16% of the simulated factor remained on the side of the chamber from which it was released, while a minimal degree of transverse diffusion resulted in the opposite side of the chamber (simulating cells not releasing this hypothetical factor) receiving an average of 0.16% of the simulated factor. The flow speeds at the withdrawal rate of 4 μl/min were also modeled within the flow module (Fig. S8e).

Computer aided design (CAD) and stereolitography files

The CAD and stereolitography files for the barrier module mold, the flow module mold and the alignment tool are included as supplementary information in the compressed folder titled Barrier_Flow_Alignment CAD and STL files.zip. The CAD files are in a '.step' format, which are can be opened and edited in standard CAD software, while the 3D printable '.stl' files are available for direct printing.

Supplementary Figure and Video Legends

Figure S1. CAD drawings (drawn with Fusion 360 software) of the barrier module mold (a), the flow module mold (b) and the alignment tool (c).

Figure S2. Effect of printing orientation on PDMS surface smoothness and printer resolution. (a) PDMS surfaces produced after casting in molds that had been printed either at an angle or parallel to the build plate of the printer. (b) Mold pillars created to test the accuracy of the printer, and formation of corresponding cavities (c) in cast PDMS chips (all units = μm).

Figure S3. Analysis of the intended and actual z-height of PDMS structures cast from 3D printed molds. (a) Illustration of a mold containing 9 cuboid structures with heights increasing in steps of 100 μm from 100 μm to 900 μm. Three replicates were 3D printed. (b) Illustration demonstrating the cross-sectional profile of the steps in the mold. PDMS chips were cast from the mold and three cross-sections (approximately 1 mm thick) were cut

through the length of each chip using a microtome blade, and mounted on a glass slide. The insert shows an example of one of the PDMS wells (900 μm z-axis dimension) from one of the cross-sections; *h* indicates the measurement taken using the ImageJ line tool to determine the actual height of the PDMS wells. (c) Representative images of all 9 wells from one cross-section cut from one PDMS chip. Numbers represent the mean and standard deviation of the actual height measurements (μm) for all replicates (i.e. n= 3 molds, and 3 cross-sections per mold); the intended height (μm) is presented in parentheses. (d) The actual height of each PDMS well for each of the 3 molds [mean ± standard deviation (dashed line)] was plotted against the intended height and linear regression analysis was performed with Prism 7. Note that the yellow data-points representing the mean height of structures intended to be 100 μm and 200 μm had a greater diameter than their associated standard deviation, thus, no dashed line is visible.

Figure S4. (a) Celltracker red fluorescence intensity represented as a 16-color spectrum (left panel) reveals that HUVEC confluence can be underestimated when visualized using single color scales (right panel) due to non-uniform endothelial cell (EC) height (*h*) and consequently low dye concentration at the edges of the cells, as illustrated by the comparison in (b). (Original magnification: (a) 20x objective; Scale bar = 20 μm.)

Figure S5. Leukocyte attachment to HUVECs in the flow module. (a) 16-color representation of signal intensity of Celltracker red fluorescence in a confluent patch of HUVECs. (b) Time-lapse images of a leukocyte decelerating and attaching within the area enclosed by the white frame in (a). The final panel in (b) is a color-coded temporal representation of the 30 s time-lapse sequence. (c) Determining the x- and y-coordinates for the leukocytes' leading edge at each time-point permits velocity analysis. (Original magnification: 20x objective; Scale bar in (a) = 20 μm. Applied flow rate: 4 μl/min).

Figure S6. Effect of vacuum bonding on the height of the flow module. (a) Fluorescent microspheres (2 μm diameter) suspended in an agarose gel (0.4%) were drawn into the flow module. Once the gel had polymerized a z-stack (1 μm optical section thickness) through a central location in the flow module with vacuum off was collected by confocal laser scanning microscopy and the distribution of fluorescent microspheres through the height of the flow channel was visualized. The identical location was imaged with the same z-stack settings with the vacuum on. Images are pseudo-colored such that fluorescent microspheres are presented as red when the vacuum was off, and green when the vacuum was on. (b) The complete set of confocal z-stacks for all images captured with the vacuum off and on were

projected into a single x, y plane using ImageJ. These z-projection images of fluorescent microspheres with the vacuum off and on were merged into a composite image, and one region is enlarged to aid in visualizing the degree of spatial overlap (Zoom). (c) To assess the distribution of fluorescent microspheres along the z-axis of the flow module with the vacuum off and on the confocal z-stacks were resliced in ImageJ and projected into a single z, x plane. These stack projections of fluorescent microspheres with the vacuum off and on were again merged into a composite image. (d) A region from the upper and lower parts of the flow chamber are enlarged to aid in visualizing the degree of spatial overlap (Zoom). Individual fluorescent microspheres in the upper (n = 35) and lower positions (n = 32) of the flow module with vacuum off and on were identified as regions of interest in ImageJ using a circular selection tool with fixed diameter, and the spatial coordinates for each were recorded. The displacement of microspheres caused by applying vacuum was calculated and presented as a percentage of the flow channel height (200 μm). (Scale bar: (b, c); Composite = 50 μm, (b); Zoom = 10 μm).

Figure S7. Leukocyte distribution in the flow module. (a) Images of Celltracker green leukocyte fluorescence from the final time-point of three independent adherence experiments were stacked into a single image. Rectangular regions of interest on the Ctrl and TNFα-treated sides of the flow module were defined from top-to-bottom along the vertical (20 regions in total) and horizontal (10 regions in total) axes of the image (indicated by white ticks). The area of leukocyte fluorescence in each region is presented as a percentage of the total area of leukocyte fluorescence from all defined regions along the vertical (b) or horizontal axes (c), respectively. Two examples of the ROIs selected on the vertical axis are illustrated by the pink- and yellow-colored frames, and one example of the ROIs selected on the horizontal axis is illustrated by the cyan colored frame.

Figure S8. Analysis of laminar flow properties in the flow module. (a) The inlet and outlets of the flow module were connected as for the leukocyte experiments and the chamber was prefilled with a black dye diluted in water, which resulted in a solution containing suspended dye particles. The center of the flow module was viewed using the live feed from the AxioVision imaging software, and recorded in real-time using screen capture software. A flow rate of 4 μl/min was applied. (b) Videos of dye particles flowing through the flow module were imported to ImageJ as image stacks and individual particles were tracked using the Manual Tracking ImageJ plugin over 100 frames. The complete track (dot-and-line) for each tracked particle is presented overlaid on frame 1, and for clarity are also presented on a black background (c). (d) A finite elements model of the flow module containing a simulated

Ctrl HUVEC population and TNFα-treated HUVEC population was constructed in COMSOL. The TNFα treated side was modeled such that it constantly released a fixed concentration of TNFα, while the Ctrl side was modeled as releasing no TNFα. A flow rate of 4 μl/min, as used in the leukocyte experiments, was applied to the model. The effects of potential transverse mixing of TNFα were evaluated by assessing cytokine distribution in the model at 25 mins (i.e. the same duration as that of the leukocyte experiments). The average concentration of simulated TNFα was 0.16% on the Ctrl side of the chamber, and 98.16% of the maximum concentration secreted from the treated side of the chamber. (e) Illustrations presenting the speed of flow in the module as modeled at the withdrawal rate of 4 μl/min at t = 1 sec and t = 25 mins. (Original magnification: 10X; Scale bar in (b) and (c) = 50 μm).

Video V1. Leukocyte attachment to HUVECs in the flow module. Time-lapse imaging (15 sec intervals) of leukocytes attaching to Ctrl (left side) and TNFα-stimulated (right side) endothelial cells. (Original magnification: 5x objective. Applied flow rate: 4 μl/min).

Video V2. Leukocyte attachment to HUVECs in the flow module. Time-lapse imaging (5 sec intervals) of leukocytes decelerating and attaching to HUVECs. (Original magnification: 20x objective. Applied flow rate: 4 μl/min).

Video V3. Manual tracking of dye particles passing through the flow module. The live feed from the AxioVision imaging software of dye particles moving through the central position of the flow module was recorded using screen capture software and imported into ImageJ. Nine particles were selected and their paths through the flow chamber were tracked for 100 frames using the ImageJ Manual Tracking plugin. The resulting dot-and-line tracks are presented as overlays on the original video (left panel), and on their own (right panel). (Original magnification: 10x objective. Applied flow rate: 4 μl/min. Video rate: 10 frames/sec).

References

1 Goodhill, G. J. Mathematical guidance for axons. *Trends in Neurosciences* **21**, 226-231, doi:https://doi.org/10.1016/S0166-2236(97)01203-4 (1998).

Figure S1

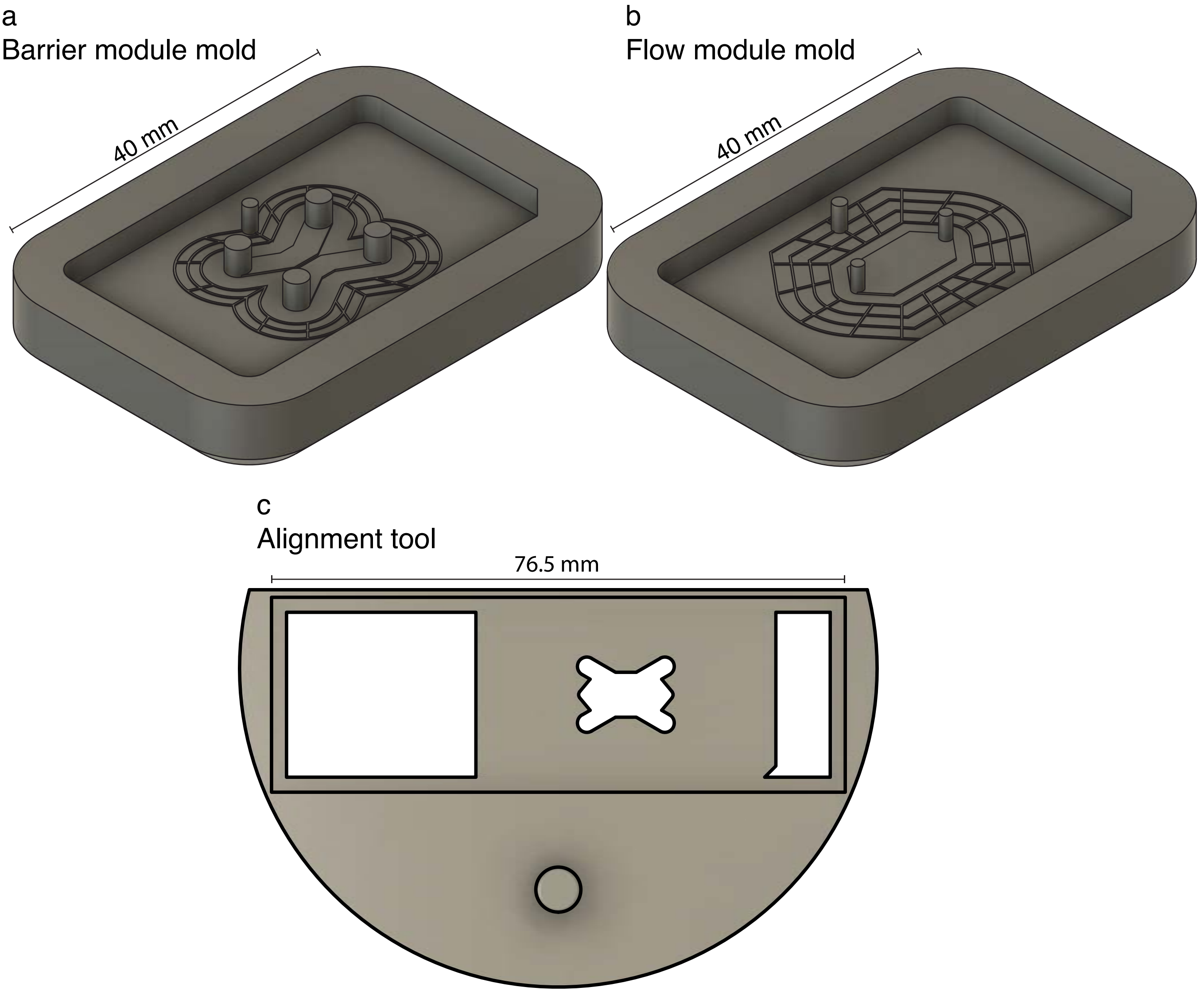
a
Barrier module mold
40 mm
b
Flow module mold
40 mm
c
Alignment tool
76.5 mm

Figure S2

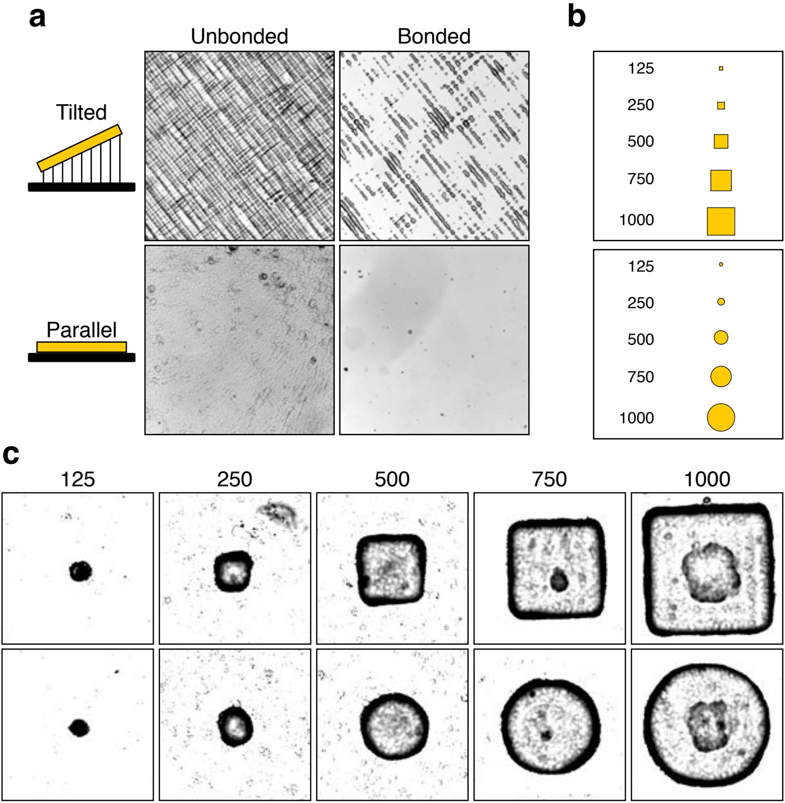
a
Unbonded
Bonded
Tilted
Parallel
b
125
250
500
750
1000
125
250
500
750
1000
c
125
250
500
750
1000

Figure S3

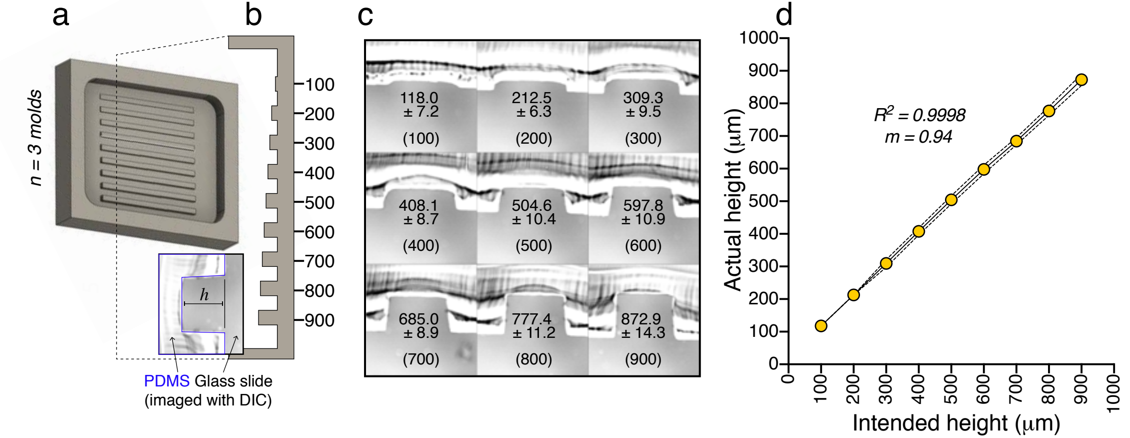
a
b
c
d
n = 3 molds
h
PDMS Glass slide
(imaged with DIC)
100
200
300
400
500
600
700
800
900
118.0 ± 7.2 (100)
212.5 ± 6.3 (200)
309.3 ± 9.5 (300)
408.1 ± 8.7 (400)
504.6 ± 10.4 (500)
597.8 ± 10.9 (600)
685.0 ± 8.9 (700)
777.4 ± 11.2 (800)
872.9 ± 14.3 (900)
R² = 0.9998
m = 0.94
Actual height (μm)
Intended height (μm)
0
1000

Figure S4

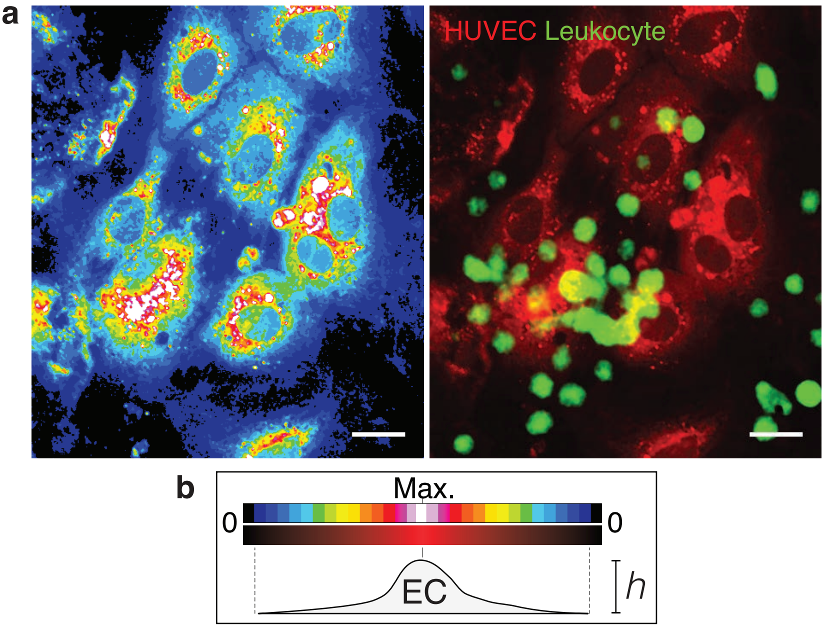

Figure S5

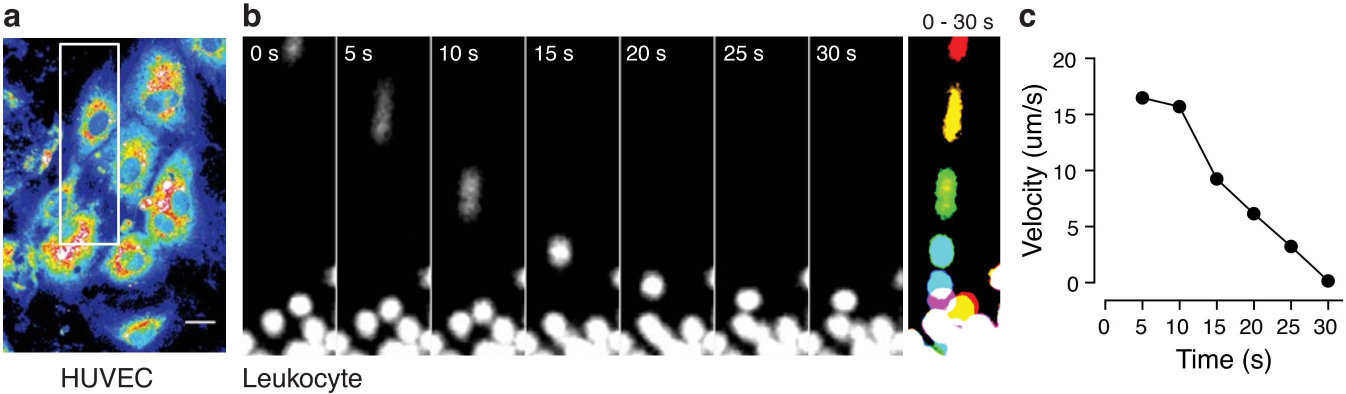
a
b
c
HUVEC
Leukocyte
0 s
5 s
10 s
15 s
20 s
25 s
30 s
0 - 30 s
Velocity (um/s)
Time (s)
0
5
10
15
20
25
30

Figure S6

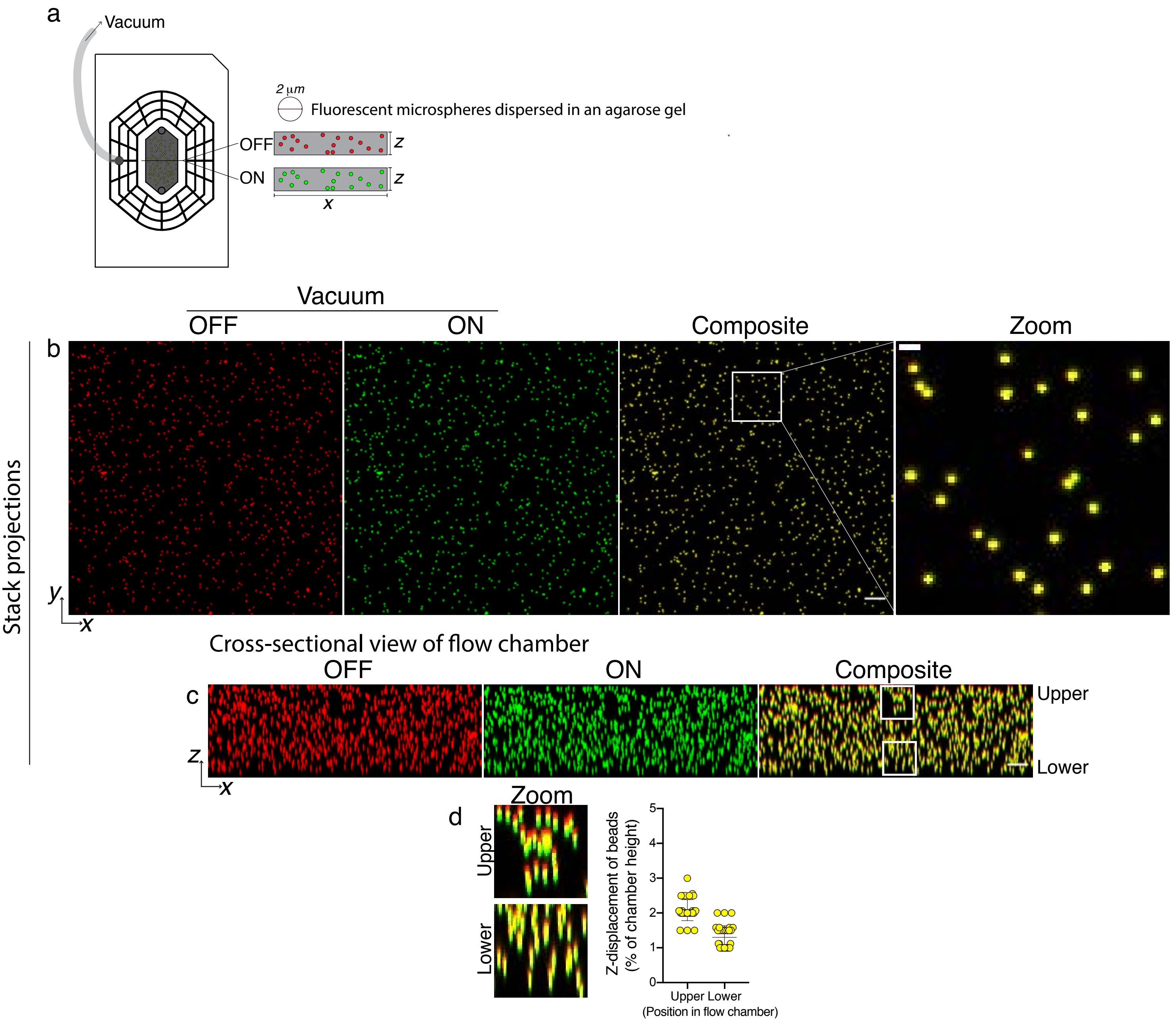
a
Vacuum
2 µm
Fluorescent microspheres dispersed in an agarose gel
OFF
ON
z
z
x
Vacuum
OFF
ON
Composite
Zoom
b
Stack projections
y
x
Cross-sectional view of flow chamber
OFF
ON
Composite
c
Upper
Lower
z
x
Zoom
d
Upper
Lower
Z-displacement of beads (% of chamber height)
0
1
2
3
4
5
Upper
Lower
(Position in flow chamber)

Figure S7

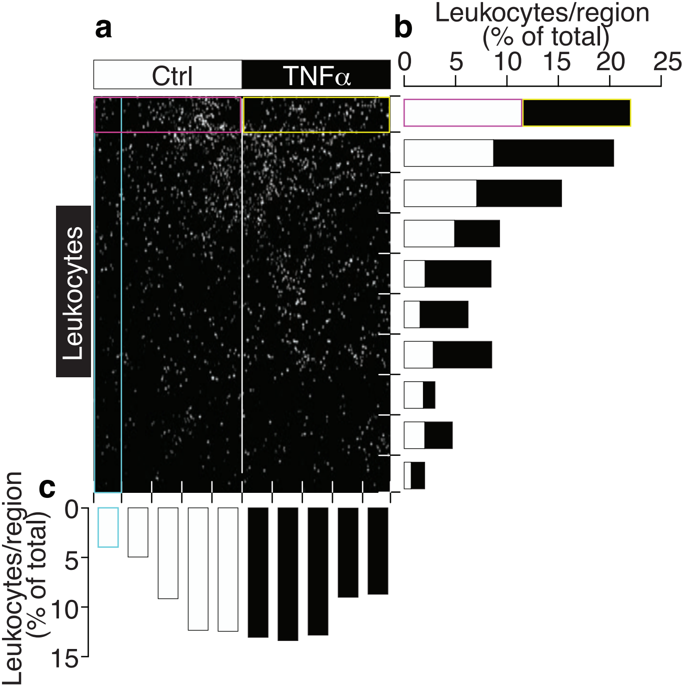
a
Ctrl
TNFα
Leukocytes
b
Leukocytes/region
(% of total)
0
5
10
15
20
25
c
Leukocytes/region
(% of total)
0
5
10
15

Figure S8

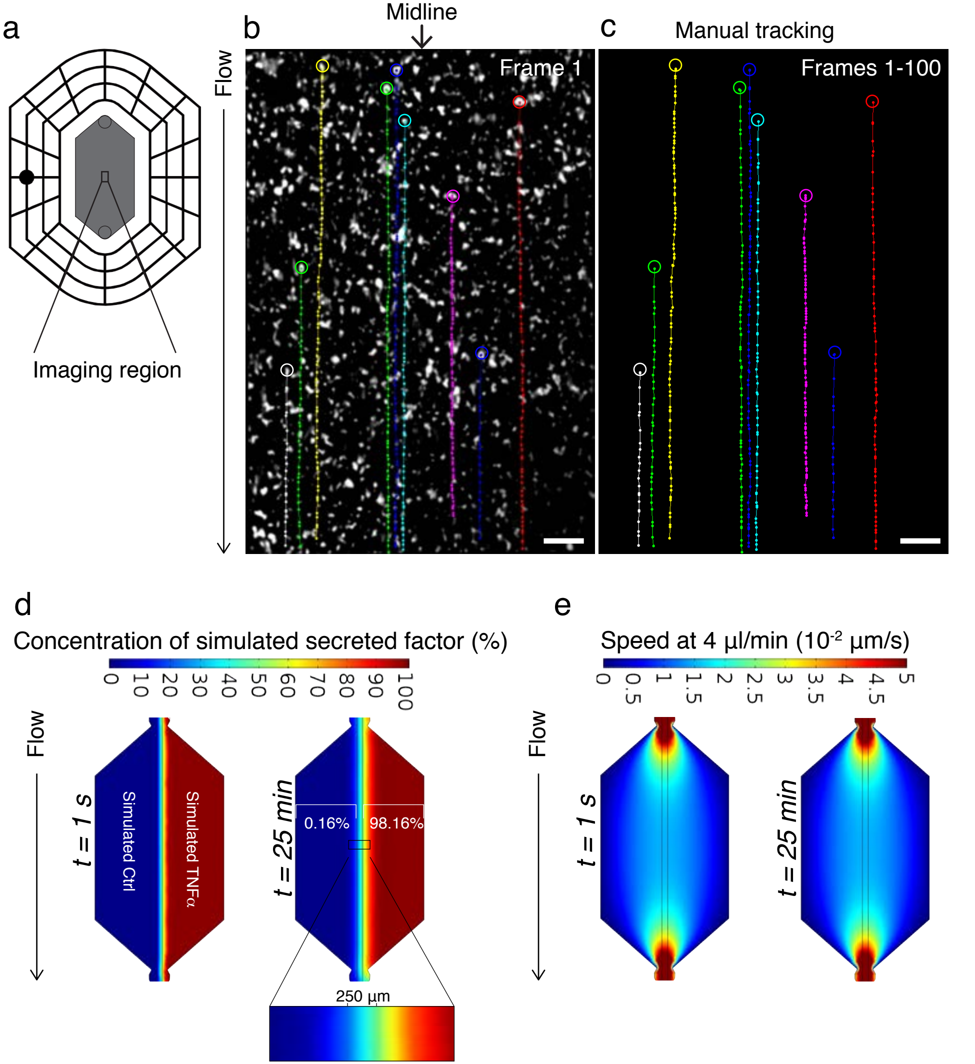
a
Imaging region
b
Midline
Flow
Frame 1
c
Manual tracking
Frames 1-100
d
Concentration of simulated secreted factor (%)
0 10 20 30 40 50 60 70 80 90 100
Flow
t = 1 s
Simulated Ctrl
Simulated TNFα
t = 25 min
0.16%
98.16%
250 µm
e
Speed at 4 µl/min (10-2 µm/s)
0 0.5 1 1.5 2 2.5 3 3.5 4 4.5 5
Flow
t = 1 s
t = 25 min
